# Effects of Different Wearable Resistance Placements on Running Stability

**DOI:** 10.3390/sports12020045

**Published:** 2024-02-01

**Authors:** Arunee Promsri, Siriyakorn Deedphimai, Petradda Promthep, Chonthicha Champamuang

**Affiliations:** Department of Physical Therapy, School of Allied Health Sciences, University of Phayao, Phayao 56000, Thailand; 63130474@up.ac.th (S.D.); 63130340@up.ac.th (P.P.); 63130104@up.ac.th (C.C.)

**Keywords:** treadmill running, weight vest, wearable resistance training, recreational runners, running gait, running stability, local dynamic stability, largest Lyapunov exponent, smartphone-based accelerometry

## Abstract

Stability during running has been recognized as a crucial factor contributing to running performance. This study aimed to investigate the effects of wearable equipment containing external loads on different body parts on running stability. Fifteen recreational male runners (20.27 ± 1.23 years, age range 19–22 years) participated in five treadmill running conditions, including running without loads and running with loads equivalent to 10% of individual body weight placed on four different body positions: forearms, lower legs, trunk, and a combination of all three (forearms, lower legs, and trunk). A tri-axial accelerometer-based smartphone sensor was attached to the participants’ lumbar spine (L5) to record body accelerations. The largest Lyapunov exponent (LyE) was applied to individual acceleration data as a measure of local dynamic stability, where higher LyE values suggest lower stability. The effects of load distribution appear in the mediolateral (ML) direction. Specifically, running with loads on the lower legs resulted in a lower LyE_ML value compared to running without loads (*p* = 0.001) and running with loads on the forearms (*p* < 0.001), trunk (*p* = 0.001), and combined segments (*p* = 0.005). These findings suggest that running with loads on the lower legs enhances side-to-side local dynamic stability, providing valuable insights for training.

## 1. Introduction

Wearable resistance training is a method that enables individuals to attach gear or garments with additional loading to various body parts [1]. Widely employed in athletic training, it aims to find optimal loads that provide effective resistance training without negatively impacting sporting technique, i.e., to explore suitable loads that allow movements to occur without unintentionally impacting the technical execution of the action [1]. This method has been applicable for various purposes, e.g., warm-ups [2], training [3], or sports activities [4]. In the context of running, wearable resistance training involves intentionally adding external resistance or weight to running exercises [1,5,6]. This results in greater ground reaction forces and increased power production and velocity during sprint running [4]. Runners employ various load-bearing strategies, e.g., weighted vests, forearm cuffs, or lower leg cuffs [6]. Trunk loading, exemplified by wearing weighted vests, allows an overload to be uniformly distributed close to a person’s center of gravity, potentially enhancing the capacity to generate higher ground response forces and power [7]. Conversely, loads applied to the distal segments of the limb are usually positioned near their end, increasing the moment of inertia and, consequently, the amount of muscular activity needed [8]. Despite these advantages, it remains unclear whether the placement of such equipment on different body parts affects running stability.

Stability in locomotion, which refers to an individual’s capacity to maintain balance and control while in motion, is widely recognized as the inherent ability of the motor control system to preserve or return to its initial state even when faced with internal factors (e.g., neuro-muscular aspects) and external perturbations (e.g., environmental) [9,10,11]. Measures of stability offer valuable insights into understanding the inherent variability in motor task performance, allowing for the direct quantification of dynamic error correction [9,10,11] since human movement is accepted to arise from non-linear interactions among various neuromuscular elements influenced by both internal and external factors [9,10]. In this sense, a non-linear analysis of human movement has been suggested [12]. For instance, the largest Lyapunov exponent (LyE), a method used to assess local dynamic stability, quantifies the exponential rate of divergence of trajectories within the state space, specifically of kinematic data acquired from gait [13,14]. In the context of locomotion stability, LyE serves to evaluate the capacity of the neuromuscular system to adapt and manage infinitesimal perturbations to sustain functional locomotion [10,15,16]. A higher LyE indicates greater complexity and unpredictability, signifying that minor differences in initial conditions lead to significant variations in movement patterns over time [14]. Conversely, a lower LyE suggests more consistent and predictable movements, where minor initial differences result in minimal variations over time [14]. Therefore, stability during locomotion encompasses a multifaceted concept, including the body’s ability to maintain balance, reduce deviations, and control movement effectively [17,18,19]. Less stability during running has been recognized as one of the major contributors to decreased running performance and increased risks of injury [20].

It is widely accepted that every aspect of human movement involves acceleration, primarily driven by muscle actions [21,22]. In maintaining a stable running posture, body accelerations result from a combination of muscle actions [23] and the intricate interplay between the neuromuscular system and other factors, such as gravity, ground reaction forces, friction, air resistance, and external forces [24]. Together, these components modulate muscle-activation levels to provide the appropriate acceleration [25]. These body accelerations essentially reflect the ability of the sensorimotor system to control the body’s motion and preserve stability throughout this dynamic activity, and they are crucial to understanding the mechanics of running [17,18]. Comprehending this idea via the application of the Lyapunov exponent (LyE) to body acceleration, as shown in earlier review studies [13,26], might be very important for running-related training, injury prevention, and rehabilitation initiatives. When considering external forces, accelerations can inadvertently manifest, especially in the presence of wearable resistance equipment, challenging running stability. Furthermore, carrying weights on different body parts may necessitate posture adjustments and enhanced neuromuscular control to preserve stability while running.

In summary, understanding how different load distribution strategies impact running stability may be pivotal for optimizing training regimens and enhancing overall running performance. Hence, the current study aimed to investigate how load distribution across various body parts—whether focused on adding the load to the trunk, lower legs, forearms, or combinations of these segments—affects local dynamic running stability, as quantified using the Lyapunov exponent (LyE) based on body acceleration. Given that applying external loads during running can lead to altered movement patterns and magnitudes [1,5,6], the hypothesis was that differences in running stability would be observed between running with no load and running with loads on different body parts.

## 2. Materials and Methods

### 2.1. Participants

Fifteen recreational male runners (age range: 19–22 years) with running exercise regularly at least 3 days/week and good experience with treadmill running participated in the study; their characteristics are represented in Table 1. All participants were university students with a normal body mass index (BMI) who had self-reported no neurological or musculoskeletal problems within the last six months, no medical conditions (e.g., diabetes, high blood pressure, heart disease, or other diseases), and no experience with wearable resistance training. In addition, runners who had wounds or pains or had a history of consuming alcoholic beverages or energy drinks within 24 h before testing were not eligible for the study. All participants were selected from the invitations via electronic flyers through online platforms and then screened through personal contact in order to provide experimental information for self-preparation before performing the running tests. 

In order to calculate the sample size of the current study, a priori power analysis through the G*Power software version 3.1.9.4 (Heinrich-Heine-Universität Düsseldorf, Düsseldorf, Germany) [27] was used based on a previous preliminary study that assessed the effects of wearable resistance placement by measuring acceleration data [27], yielding an average effect size for comparisons between the placement conditions of 0.42. Based on this computation, with a significance level of a = 0.05 and a desired power of 0.95, the suggested sample size was N = 14. However, fifteen young adults volunteered to participate in the current study. The current experimental procedures were approved by the Institutional Review Board of the University of Phayao, Thailand (Approval Code No.: HREC-UP-HSST 1.3/038/66, Approval Date: 20 August 2023) and conducted in accordance with the Declaration of Helsinki. All volunteers provided written, informed consent before participating.

### 2.2. Equipment and Experimental Procedure

On the day before the experiment, each participant was asked to complete a questionnaire to self-check their health status and was informed to prepare appropriate clothing and shoes for running, obtain enough rest (at least 6–8 h), eat a meal at least 2–3 h before the test, refrain from consuming alcohol or energy drinks for at least 24 h, and avoid strenuous exercise for at least 24 h.

On the experimental day, all participants were asked to check their blood pressure and be screened by the researcher for any wounds or injuries. In the current study, ref. [28] a tri-axial accelerometry sensor embedded in a smartphone (Samsung Galaxy A52s 5G, Samsung Electronics Co., Ltd., Suwon, Republic of Korea) was employed to measure trunk acceleration [28]. As previously reviewed, smartphone-based accelerometry has been accepted as an alternative, valid, and reliable measure of gait and postural control [28,29]. The smartphone was securely positioned using a waist-mounted pouch in the lumbar region (L5), which was close to the body’s center of mass [30,31]. The accelerometer functioned using the Physics Toolbox Sensor Suite application (version 2023.01.07) on the Google Android platform [32,33], enabling the collection and export of acceleration data at a sampling rate of 200 Hz. All participants wore their running shoes for all study runs. Minimalist running shoes were not allowed due to their effects on increased vertical loading rates compared to cushioned running shoes [34]. 

**Figure 1:**
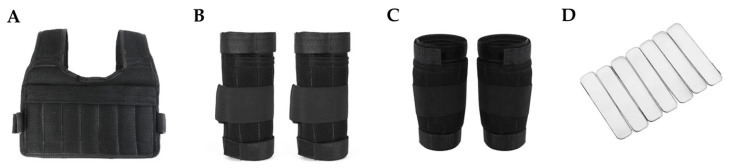
Wearable resistance equipment ((**A**): a weighted vest, (**B**): forearm cuffs, (**C**): lower leg cuffs, and (**D**): detachable metal plates).

All participants began the study with a warm-up consisting of a 5 min brisk walk on a Brightway TT-X10 treadmill (Shandong Brightway Fitness Equipment Co., Ltd., Shandong, China) at a speed of 5.5 km/h and a 5 min whole body stretching [35], and then rested for 5 min before the first experiment ran. Subsequently, volunteers were asked to complete five treadmill running trials in random order: no-load running and running with a load equivalent to 10% of their body weight placed on their lower arms, lower legs, trunk, and a combination of forearms, lower legs, and trunk. The choice of a 10% load of individual body weight was based on its effectiveness in improving running performance, as reported in the systematic reviews [1,6,8]. The load was applied using a weighted vest, forearm cuffs, and lower leg cuffs inserted via the detachable metal plate (Figure 1). In conditions where the load was distributed across the forearms, lower legs, and combined segments, equal weight was allocated to each segment. The weight distribution was symmetrical for the anterior and posterior sides of the weight vest and evenly distributed for the forearm and lower leg cuffs. 

During testing, if the participant had any abnormal symptoms during the test, e.g., dizziness, nausea, vomiting, pain, or accidents, it would be considered a withdrawal of participant criteria, and they could also request to terminate their participation if they felt unsafe or did not wish to participate in the research.

For each running condition, the treadmill’s speed gradually increased from 0 to 10 km/h within 1 min, remained constant for 3 min, and gradually decreased from 10 to 0 km/h within 1 min. The selected running speed of 10 km/h is closely related to the preferred running speed of recreational runners [18]. Each test consisted of 5 min of running per condition, with participants allowed a 5 min rest period after each test. Borg’s rating of perceived exertion (RPE) scale was used to measure perceived exertion or exercise intensity at the baseline period (before performing experiments) and after each running test [36,37]. The Borg RPE scale used in this study ranges from 6 to 20, with 6 representing very light exertion and 20 representing maximum exertion or exhaustion [36,37]. Since the scale is subjective, individuals rate their own perceived exertion based on their feelings of effort, fatigue, and other sensations during exercise [36,37]. In addition, before starting any running trial, all volunteers were asked to check their readiness; if the 5 min rest was not enough, they could stay for a longer rest. All participants attended one testing session in a temperature-controlled (25 °C) laboratory. The acceleration signals during the 3 min treadmill run for each running condition were recorded using a Samsung Galaxy Tab S6 Lite tablet (manufactured by Samsung Electronics Co., Ltd., Suwon, Republic of Korea) to operate the Physics Toolbox Sensor Suite application installed in the smartphone using the Samsung Flow application version 4.9.08.3 (Samsung Electronics Co., Ltd., Suwon, Republic of Korea).

### 2.3. Data Analysis

**Figure 2:**
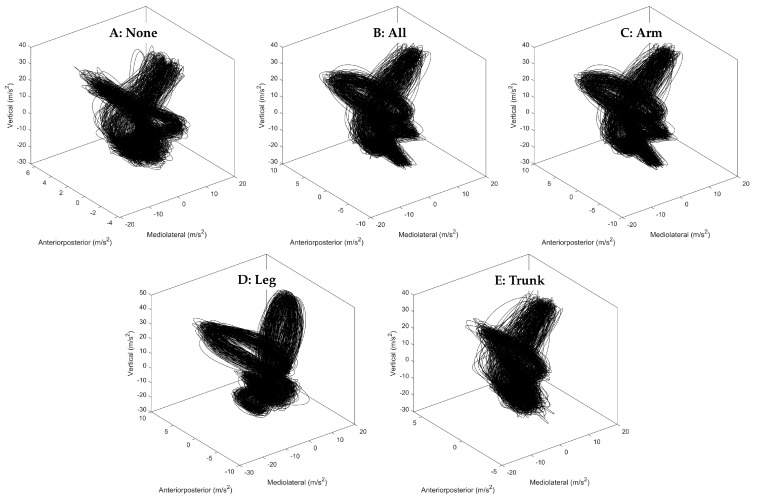
Example tri-axial acceleration data of the treadmill running with (**A**) no load, (**B**) and running with loads on combined (forearms, legs, and trunk) segments, (**C**) forearms, (**D**) lower legs, and (**E**) trunk, respectively. Note: the presented middle 2 min tri-axial acceleration data were retrieved from each running condition of the first participant.

MATLAB^TM^ (MathWorks Inc., Natick, MA, USA) was used for all data processing. A Fourier analysis was applied to the raw acceleration signals, revealing that the highest power was concentrated in frequencies around 5–10 Hz, with visible power still present in the 15–20 Hz frequency range. Consequently, these signals underwent smoothing through a 4th-order zero-phase 20 Hz low-pass Butterworth filter, similar to that previously reported [38]. For the analysis of acceleration-based variables, the middle two minutes of each acceleration signal (anteroposterior (AP), mediolateral (ML), and vertical (VT) accelerations) were selected to exclude any movements associated with adjustments to the desired running speed. Figure 2 provides an example of visual representations of the smoothed tri-axial acceleration data during running with each condition.

**Figure 3:**
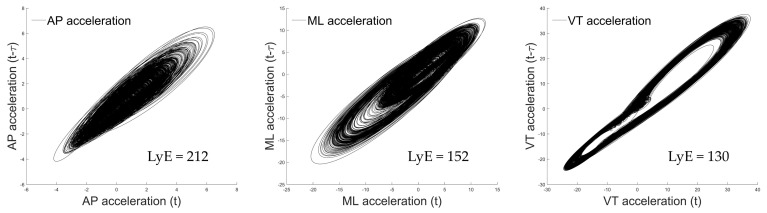
Example of the space–time representation for the calculated largest Lyapunov exponent (LyE) of a tri-axial acceleration data of running with no load. Note: the presented data were derived from the first participant.

Then, two acceleration-based variables were computed for each acceleration signal. First, the largest Lyapunov exponent (LyE) was applied to individual acceleration data [14] to investigate running stability by calculating the rate of divergence of closely related trajectories in a state space during locomotion [17,18,19,39]. This metric offers insights into the motor system’s ability to attenuate minor perturbations, and LyE was computed using Wolf’s algorithm [40]. The parameters for LyE calculation, including time delay (τ = 10) and embedding dimension (m = 4), were determined using the average mutual information (AMI) and the false nearest-neighbor algorithms [17,18,19,39]. A higher LyE value indicates a reduced ability of the motor system to counter infinitesimal perturbations [15], ultimately resulting in greater divergence of state space trajectories and, by extension, lower stability during locomotion [12,41]. Figure 3 shows an example of the space–time representation for the calculated LyE of a tri-axial acceleration. 

Second, the root-mean-square (RMS) was employed as a measure of the magnitude or intensity of an acceleration signal [42]. This metric serves to evaluate the forces and stresses imposed on the body during physical activities.

### 2.4. Statistical Analysis

All statistical analyses were performed using the SPSS software version 26.0 (IBM SPSS Statistics, SPSS Inc., Chicago, IL, USA), with the alpha level set at α = 0.05. A Shapiro–Wilk test was used to test the normal distribution of the considered variables. A one-way repeated-measures ANOVA was used to test the effects of running conditions. The effect sizes (Partial Eta Square; η_p_^2^) and observed power (1 − β) were also reported. To adjust the alpha level to control the familywise error rate with five running conditions, the alpha level of post hoc analysis was set at α < 0.005.

## 3. Results

All participants could complete all running tests without any discomfort and did not meet the withdrawal criteria. Regarding the measure of rating of perceived exertion, the average scale of baseline or the rest before experiments is 6.6, representing the feeling of very, very light of perceived exertion, while after each running condition, the average scale is 11.0 for running with no load (None) and 11.5, 11.9, 10.9, and 11.5 for running with loads on combined segments (All), forearms (Arm), lower legs (Leg), and trunk (Trunk), respectively, indicating the feeling of fairly light of perceived exertion for all types of running with load conditions.

**Figure 4:**
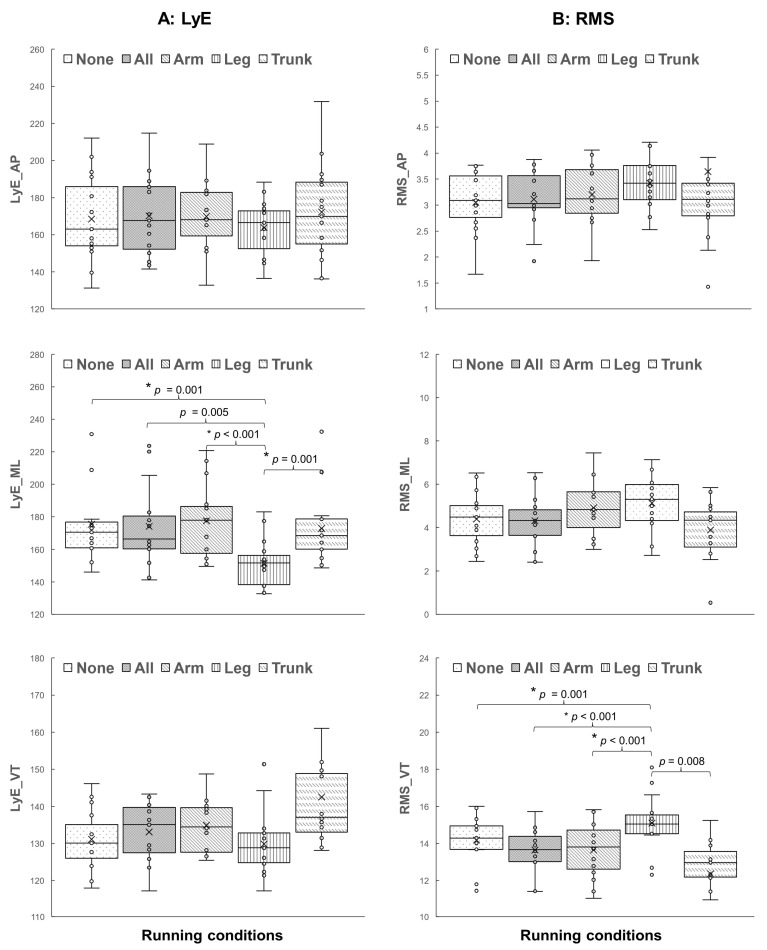
The post hoc comparisons of (**A**) the largest Lyapunov exponent (LyE) and (**B**) the root-mean-square (RMS) between five running conditions: running with no load (None) and running with loads on combined segments (All), forearms (Arm), lower legs (Leg), and trunk (Trunk) (* *p* < 0.005).

Regarding running stability, the results show that the main effects of different wearable resistance placements on local dynamic stability assessed by the LyE are only observed in specific acceleration directions. Specifically, load distribution effects appear only in the mediolateral direction, LyE_ML (F_(2.46, 34.39)_ = 11.10, *p* < 0.001, η_p_^2^ = 0.442, 1 − β = 0.995). The post hoc tests (Figure 4) reveal that running with the load on lower legs has a lower LyE_ML value than running with no load (*p* = 0.001), running with the load on combined segments (*p* = 0.005), running with the load on forearms (*p* < 0.001), and running with the load on the trunk (*p* = 0.001).

Moreover, the main effects of different load distributions on running magnitude assessed using RMS variables are only observed in vertical acceleration, RMS_VT (F_(1.21, 16.93)_ = 10.08, *p* = 0.004, η_p_^2^ = 0.419, 1 − β = 0.889). The post hoc tests (Figure 4) reveal that running with the load on lower legs has a greater RMS_VT value than running with no load (*p* = 0.001), running with the load on combined segments (*p* < 0.001), running with the load on forearms (*p* < 0.001), and running with the load on the trunk (*p* = 0.008).

## 4. Discussion

The present study investigated the effects of load distribution across various body parts by focusing on adding external loads to the forearms, lower legs, trunk, and combinations thereof, and no load on running stability was assessed via body acceleration. Two acceleration-based variables, the largest Lyapunov exponent (LyE) and the root-mean-square (RMS), were computed for each acceleration signal to measure local dynamic stability and the magnitude of running movements, respectively. The primary findings indicate that load distribution effects exist in both variables but are specific to certain acceleration directions. Notably, the effects on local dynamic running stability (LyE) and movement magnitudes (RMS) are observed in the mediolateral (ML) and vertical (VT) acceleration directions, respectively. Considering the current empirical findings, two key points can be discussed. 

First, significant load distribution effects on local dynamic running stability, as observed in the mediolateral direction (LyE_ML), suggest that adding load to the lower legs primarily influences a runner’s side-to-side stability during treadmill running. Since the LyE is a measure of the system’s sensitivity to initial conditions and the predictability of motion [14], the lower LyE value in this direction indicates less chaotic and more predictable side-to-side movements when using lower leg resistance loads. In other words, the added resistance on the lower legs may stabilize and reduce the variability in the runner’s side-to-side motion as a stabilizing force, potentially helping the runner maintain a more controlled and consistent stride width during running. However, for a more comprehensive understanding of these stability effects, it is essential to delve into biomechanical intricacies. For example, limb inertia, representing resistance to changes in motion [43,44], may vary based on load placement, influencing overall running dynamics. Changes in limb inertia, combined with load distribution, could contribute to nuanced alterations in side-to-side stability during treadmill running. While the study did not explicitly measure limb inertia, recognizing its potential influence adds complexity to the interpretation of the results. In addition, it has been reported that wearable loads could alter the step variables, although the treadmill speed is fixed [1]. Future investigations should consider examining limb inertia or step variables with different load placements to disentangle their contribution, which is of interest. Although the specific mechanisms influencing this running stability related to externally added loads are unclear, it can be assumed that running with loads placed on the lower legs may challenge the sensorimotor system, specifically through directly increased muscle activation in the lower limbs [8]. Previous studies suggest that performing challenging lower extremity tasks can be seen as a facilitation for the neuromuscular control of the lower limb muscles, which are the main group muscles in performing locomotion tasks, possibly by increasing myoelectric activity [22], muscular output [45], and inter-muscular coordination [46]. 

Second, concerning the magnitude of movements, a higher RMS value in the vertical direction (RMS_VT) indicates that the addition of resistance loads to the lower legs leads to an increased intensity of vertical motion. This effect can be attributed to the inherent bouncing motion associated with running [47]. Running is often likened to a bouncing motion, where kinetic and gravitational potential energy is momentarily stored as elastic strain energy components, including muscles, tendons, and ligaments, upon foot strike [47]. Subsequently, these energies are recovered during the propulsive phase of the stance [47]. In simpler terms, with each step, mechanical energy is absorbed as the body decelerates during the braking phase and released as the body reaccelerates during the push-off phase [48]. The addition of resistance to the lower extremities, as shown in previous studies, has been found to increase joint reaction forces and net moments of force compared to running without added loads [45]. This heightened intensity can be attributed to the increased force required to lift the legs against gravity due to the bouncing effect [47]. The current finding presents potential advantages and disadvantages. In the context of strength and power development, a higher RMS_VT may be considered beneficial. It suggests that runners are working against gravity to a greater extent, potentially leading to improved lower-limb strength. Athletes in specific sports, such as sprinting, may intentionally aim for higher RMS_VT values due to their relevance to their specific training needs and competitive performance [5,8]. On the other hand, excessive vertical oscillation, characterized by up-and-down motion, may lead to increased energy expenditure, fatigue, and slower running times, of which minimizing vertical motion is often a goal for efficient long-distance running [49]. A high RMS_VT could also imply greater impact forces on each stride, which may increase the risk of overuse injuries, e.g., shin splints or stress fractures [50]. 

From a practical perspective, the use of resistance loads on the lower legs can alter a runner’s kinematics by enhancing mediolateral stability while increasing vertical movement magnitude. The choice of resistance load placement should align with the runner’s specific training goals. For instance, controlled and stabilized mediolateral movement may help to reduce the risk of injuries related to excessive side-to-side motion, promote more efficient and controlled gait mechanics, and enhance strength and power development. Moreover, since acceleration in this study was measured closely to the body’s center of mass [30,31], it could also be inferred that the use of lower leg resistance loads encourages runners to engage core muscles and improve mediolateral balance. However, it is important to note that adding loads to the lower legs implies greater impact forces on each stride, which may elevate the risk of overuse injuries. Appropriate footwear that can absorb shock is essential [51]. Furthermore, it is crucial to apply resistance loads carefully and progressively to avoid overtraining or injury [50]. However, it should be noted that the differences between treadmill and overground running biomechanics [52] warrant careful consideration when applying the current findings to the track.

### Limitations

The current study encountered several significant limitations. First, there is an inadequate exploration of the biomechanical aspects of running, specifically the type of support and individual footprints [53] for load distribution, posing a significant constraint on the study’s comprehensiveness. Second, the study does not delve into variations in outcomes across different training sessions for each running condition. This omission raises concerns about potentially diverse results under varying circumstances. Third, the focus on neuromuscular control with wearable resistance lacks electromyographic (EMG) data, limiting the examination of activation bursts [54] and necessitating caution in result interpretation. Fourth, the participants were only engaged in running for 5 min, with 3 min at a stable speed. This short duration may introduce limitations in capturing potential variations or effects, and differences in results might emerge with a more extended running period, e.g., 15 min. Fifth, all participants ran at a fixed speed rather than a percentage of their maximum speed. This fixed-speed protocol may not fully capture individual variations in running capabilities and intensity. A protocol incorporating a percentage of participants’ maximum speed could provide a more dynamic and personalized approach, offering insights into how different running intensities may interact with wearable loads. Furthermore, the exclusive inclusion of individuals with a normal body mass index (BMI) may restrict the generalizability of the findings to a broader population. Exploring the impact of wearable loads across a wider BMI spectrum could offer a more nuanced understanding of biomechanical responses since a greater BMI could affect plantar pressure during running [55]. Additionally, the diverse nature of races among amateur runners introduces potential limitations, as outcomes may be influenced by varied training backgrounds. The participants’ amateur status [56] and lack of running experience further add to the limitations, affecting running styles and generalizability. Lastly, the absence of kinetic parameters and other biomechanical data hampers a comprehensive analysis, underscoring the need for their inclusion in future research to better grasp the implications of wearable loads on running stability.

## 5. Conclusions

Investigating the effects of different wearable resistance placements on local dynamic stability and movement magnitudes during treadmill running reveals that load distribution has distinct effects on local dynamic running stability in the mediolateral direction and the magnitude of vertical motion. Specifically, load placement on the lower legs enhances side-to-side stability while simultaneously increasing vertical acceleration displacement during running. These findings suggest that if the goal of using wearable resistance training is to promote stability, loads placed on the lower legs are recommended. However, it should also be recognized that there are potential increased impact forces, which may necessitate appropriate footwear and careful, progressive load application.

## Figures and Tables

**Table 1:** Characteristics of participants (mean ± SD).

	Max	Min	Mean	SD
Age (years)	22.0	19.0	20.3	1.0
Mass (kg)	77.0	53.0	64.7	6.5
Height (cm)	180.0	163.0	170.3	5.6
Body mass index (kg/m^2^)	26.6	19.2	22.3	2.1
Weekly mileage (km)	40.0	5.0	20.1	11.1

## Data Availability

The data presented in this study are available on request from the corresponding author. The data are not publicly available due to privacy and ethical restrictions.
